# Supplementary material for: WordCommentsAnalyzer: A windows software tool for qualitative research
Source: F1000Res. 2018 Sep 4;7:536. Originally published 2018 May 3. [Version 2] doi: 10.12688/f1000research.14819.2 (PMC6137411; doi:10.12688/f1000research.14819.2)
Supplement: Supplementary file 3 [file f1000research-7-17614-s0002.tgz › b2a79496-4ada-47c4-9682-a99ce867836d.zip!/Eight Iranian Health Journals/Yafteh _49.docx]

© Yafteh  . This work is licensed under CC BY 4.0 (https://creativecommons.org/licenses/by/4.0/).

Volume & Issue: دوره 8 - شماره 4

Title: مقایسه تأثیر اسکراب دست با بتادین و دکوسپت بر شمارش میکروبی

http://yafte.lums.ac.ir/article-1-1035-fa.html

مقدمه: براساس لزوم استفاده از روش های جدید کنترل بیماریهای واگیر و کاهش عفونتهای بیمارستانی، امروزه در کشورهای پیشرفته، اسکراب جراحی تیم استریل اتاق عمل در مدت کوتاه با مواد مؤثر، جایگزین سایر روشهای سنتی گردیده است. درحالیکه، در بیشتر اتاق عملهای کشور ما هنوز از روش سنتی و معمول اسکراب دست(بابتادین و مدت بیش از 3دقیقه) استفاده می شود. هدف از این کارآزمایی بالینی، بررسی تأثیر بتادین و دکوسپت بر میکروارگانیسم های پوست بود. مواد و روش ها: این مطالعه در اردیبهشت ماه سال1385 بر روی 20 نفر پرستار اتاق عمل بیمارستان تأمین اجتماعی شهرخرم آباد انجام شد. افراد به روش نمونه گیری مبتنی بر هدف با توجه به وجود معیارهای موردنظر انتخاب شدند. ابتدا همه افراد دستها را با صابون غیرآنتی باکتریال شسته و خشک کردند. جهت تعیین معیار پایه از دست آنها نمونه گیری در محیط TSB انجام گرفت. سپس، در دو مرحله (بعد از اسکراب با بتادین و یک هفته بعد، با دکوسپت)، جهت تعیین تأثیر فوری و دوام تأثیر دو ماده، بلافاصله بعد از اسکراب دستها با بتادین و دکوسپت و همچنین دو ساعت بعد از پوشیدن و خروج دستکش، از نوک انگشتان افراد، نمونه گیری به عمل آمده و کلنی های رشدیافته در نمونه های کشت شده شمارش شدند. آنالیز اطلاعات با نرم افزار آماریSPSS نسخه 12 انجام گرفت. باتوجه به توزیع نرمال متغیرهای نمونه های تحت بررسی، از آزمون T دوتایی برای مقایسه تفاوت میانگین تعداد کلنی های رشدیافته در هر مرحله استفاده شد. بحث و نتیجه گیری: به طور کلی، مقایسه نتایج حاصله از کشت نمونه های دست افراد تحت بررسی در دو مرحله (اسکراب با بتادین و دکوسپت) چه در رابطه با تأثیر فوری و چه دوام تأثیر آنها مؤید این بود که ماده الکلی دکوسپت بطور مشخصی تأثیر بیشتر بر کاهش میکروارگانیسمها دارد. ما پیشنهاد می کنیم که جهت اسکراب جراحی به جای سایر مواد، بیشتر از مواد الکلی استفاده شود. یافته ها: مقایسه فراوانی کاهش کلنی باکتریها بلافاصله بعد از اسکراب با بتادین ودکوسپت تفاوت داشت. مقایسه تفاوت میانگین کاهش کلنی باکتریهای پوست بلافاصله پس از اسکراب با بتادین و دکوسپت اختلاف آماری معنی داری داشت (011/0p=). همچنین، مقایسه میانگین افزایش کلنی باکتری ها از زمان اسکراب تا دو ساعت بعد از پوشیدن دستکش در دو مرحله متفاوت بود (023/0p=). رشد کلنی باکتری ها بعد از اسکراب (از بلافاصله تا دو ساعت بعد) با بتادین، نشاندهنده افزایش 7/1 بود. در حالیکه، این افزایش رشد کلنی باکتری ها در مرحله اسکراب با دکوسپت 15/1 بود که در دو مرحله تفاوت معنی داری مشاهده شد (001/0p=).

Title: تأثیر روی در بهبود اسهال حاد کودکان در شهرستان الیگودرز

http://yafte.lums.ac.ir/article-1-1036-fa.html

مقدمه: روی برای متابولیسم اسیدهای نوکلئیک،ساخت پروتئین و ترمیم بافتی ضروری است.کمبود روی به اثرات سوء بر سلامت کودک و افزایش مرگ و میر به دنبال ابتلاء به پنومونی یا گاستروآنتریت می شود. روی با افزودن ظرفیت جذبی سلولهای پرزی و کاستن باکتریهای گرم منفی پاتوژن لومن روده و مهار ترشح کلر سبب بهبود سریعتر اسهال در کودکان می گردد. مطالعات متعدد در ممالک در حال توسعه از تأثیر روی در اسهال کودکان موجودند.در مطالعه فعلی اثر بخشی روی در اسهال حاد کودکان در استان لرستان بررسی گردید. بحث و نتیجه گیری: سولفات روی می تواند به ویژه در کودکان دچار سوء تغذیه در زمان ابتلاء به گاستروآنتریت حاد به عنوان بخشی از برنامه تغذیه ای در کنار سایر دستورات تغذیه ای و بهداشتی تجویز گردد. یافته ها: دفعات اسهال در روز در گروه مورد و شاهد به ترتیب99/0± 3/3 و 1/1±9/4 بار روزانه،طول کشیدن اسهال 2/3±9/4 در برابر9/3±8/7روز و وزن گیری پس از یکماه در دو گروه به ترتیب 05/223±8/110در برابر 4/106+-8/95 گرم بود که در هر سه مورد با 05/0p< کارآیی روی در بهبود نتیجه اثبات گردید. در گروه مورد افت وزن یا اسهال پایدار دیده نشد. مواد و روش ها: این مطالعه یک کارآزمایی بالینی سه سوکور تصادفی شاهددار در الیگودرز در سال 84 است که در دو گروه مساوی 36 نفره کودکان 12 تا 60 ماهه مبتلا به اسهال حاد آبکی که شاخص وزن بر حسب قد و بر اساس تقسیم بندی واترلو،کمتر از SD2- داشتند انجام شد. در گروه مورد، روی به صورت محلول یک درصد سولفات روی به میزان یک میلی گرم به ازای هر کیلوگرم وزن بدن در روز تجویز شد. مدت زمان اسهال،تعداد دفعات اسهال در روز و افزایش وزن پس از یکماه در دو گروه مقایسه و یافته ها با نرم افزار SPSS نسخه 13 پردازش شد.

Title: بررسی میزان تراکم استخوان در افراد سالم مراجعه کننده به مرکز تراکم استخوان شهرستان اهواز در سالهای 81- 79

http://yafte.lums.ac.ir/article-1-1037-fa.html

مقدمه: استئوپوروز یکی از معضلات بهداشتی در زنان بعد از منوپوز و افراد مسن می باشد. اندازه گیری تراکم استخوان در مناطق فقرات کمری و گردن فمور یکی از موارد مطمئن و دقیق اندازه گیری توده استخوانی است و لذا می تواند ریسک شکستگی را مشخص کند. DEXA نوعی سیستم اندازه گیری غیر تهاجمی و دقیق، تراکم مغز استخوان با ضریب خطای کم و بدون عارضه می باشد. مواد و روش ها: اندازه گیری میزان تراکم استخوان بوسیله دستگاه لونار به روش دکسا صورت گرفته است. از مناطق مختلف شهر اهواز حدود سیصد فرد سالم بین سنین 20 تا 69 سال زن و مرد جهت دانسیتومتری و بعد از پرکردن پرسشنامه و اطمینان از فقدان بیماری احتمالی زمینه ای به مرکز دانسیتومتری مراجعه و از آنها اندازه گیری تراکم استخوان بعمل آمد. یافته ها: دانسیته استخوان این افراد در ستون فقرات بطور متوسط 5/5% (5/1 تا 18% ـ 1CI=) برای زنان و 2/12% (5/1 تا 27% ـ 50/1CI=) برای مردان کمتر از استاندارد لونار می باشد. در مورد دانسیته استخوان گردن فمور بطور متوسط 80/4% (5/1 تا 15% ـ 96%CI=) برای زنان و 5/8% (1 تا 19% ـ 97%CI=) برای مردان کمتر از استاندارد لونار می باشد.بحث و نتیجه گیری: بین زنان و مردان از لحاظ میانگین تراکم استخوان در گردن فمور و ستون فقرات تفاوت معنی داری در نمونه مشاهده گردید. همچنین تفاوت معنی داری بین میانگین تراکم استخوان در دو ناحیه مورد بحث در نمونه پژوهش و استاندارد لونار در گروه جنسیتی زنان مشاهده گردید. در گروه مردان تفاوت به سطح معنی داری نرسیده است.

Title: نقش مردم در مدیریت مواد زاید جامد شهری در شهر خرم آباد در سال 1384

http://yafte.lums.ac.ir/article-1-1038-fa.html

مقدمه: مدیریت مواد زائد عبارتست از مجموعه‌ای از مقررات منسجم و هماهنگ در زمینه کنترل تولید، ذخیره و یا جمع آوری، حمل و نقل، پردازش و دفع مواد زائد جامد که منطبق بر بهترین اصول بهداشت عمومی، اقتصاد، حفاظت از صنایع، زیبا شناسی و سایر الزامات زیست محیطی و مطلوب‌های عمومی است. هدف از این مطالعه بررسی میزان آگاهی و تعیین میزان مشارکت شهروندان خرم آباد در مدیرت دفع مواد زائد جامد بوده است. مواد و روشها: این مطالعه در 8 محله مختلف شهر خرم آباد به صورت پرسشنامه‌ای انجام شد. در پرسشنامه تنظیم شده مواردی مانند شرایط جمع آوری و نگهداری زباله، خطرات ناشی از جمع آوری و نگهداری زباله، خطرات ناشی از جمع آوری و دفع نادرست زباله و همچنین باز یافت زباله مورد بررسی قرار گرفت. در هر منطقه50 خانوار به صورت تصادفی ساده انتخاب گردید (جمعاً400 پرسشنامه). و پرسشنامه‌ها به آنها داده شد. بحث و نتیجه گیری: این تحقیق نشان می‌دهد که در مناطقی که از لحاظ اقتصادی و اجتماعی شرایط بهتری دارند میزان اطلاع آنها از خطرات زباله و آشنایی با واژه بازیافت بیشتر بوده است. ولی به علت آموزش کم در تمام این مناطق مسئله جداسازی در منبع تولید زباله انجام نمی شود و به همین علت هم میزان مشارکت مردم در مدیریت مواد زائد جامد بسیار ضعیف است. یافته ها: نتایج بدست آمده نشان می‌دهد که در همه محله‌های مورد نظر کلیه خانواده‌ها از کیسه زباله برای نگهداری زباله‌های خود استفاده می‌کنند همچنین عمل جمع آوری به طور روزانه در شهر خرم آباد انجام می‌شود. ولی در هیچ کدام از این محله‌ها عمل جداسازی زباله در منزل انجام نمی‌شود. همچنین میزان اطلاع افراد از محل دفع زباله و اینکه زباله بعد از جمع آوری به کجا منتقل می‌شود خیلی ضعیف است. در این مناطق بین 33 تا 70 درصد مردم اطلاع کامل از خطرات ناشی از جمع آوری و دفع نادرست زباله دارند. درصد بسیار کمی از مردم در حد 20-5 درصد با واژه بازیافت آشنا هستند. درصد رضایت مردم شهر از عملکرد شهرداری در خصوص مدیریت زباله نیز در حد 60-11 درصد می باشد.

Title: بررسی میزان وعلل دفورمیتی ها در اندام های فوقانی و تحتانی نوجوانان پسر و دختر 11 تا 15 ساله استان لرستان

http://yafte.lums.ac.ir/article-1-1039-fa.html

مقدمه: هدف از این تحقیق بررسی میزان ناهنجاری های جسمانی در بین نوجوانان پسر و دختر استان لرستان است. بحث و نتیجه گیری: نتایج تحقیق نشان می دهد که سطح و میزان ناهنجاری های جسمانی در بین پسران و دختران مورد تحقیق بسیار بالاست بنابراین نیاز به یک برنامه ریزی جدی در خصوص ناهنجاری ها و به خصوص تجویز حرکات اصلاحی و فراهم آوردن زمینه های فعالیت بدنی آنان امری لازم و ضروری به نظر می رسد. یافته ها: با توجه به یافته های به دست آمده 67 /57 درصد از پسران و 89/68 درصد از دختران دچار ناهنجاری جسمانی بوده اند. ضمن این که بین عادات خوابیدن و لوردوز کمری و کیفوز پشتی، بین طریقه حمل کیف و افتادگی شانه ها، بین طریقه نشستن چهار زانو و پای پرانتزی و همچنین بین استفاده از کفش های پنجه باریک وپاشنه بلند با شست کج ارتباط های معنی داری مشاهده گردید. مواد و روشها: جامعه آماری تحقیق را کل نوجوانان استان لرستان تشکیل می دهند که از این جامعه تعداد 400 نفر پسر و 450 نفر دختر با دامنه سنی 11 تا 15 ساله به عنوان نمونه تحقیق و به صورت تصادفی انتخاب گردیدند. اطلاعات با استفاده از پرسش نامه و آزمون های عملی همچون صفحه شطرنجی، متر، ترازو و …جمع آوری گردید. بعد از اتمام تحقیق نتایج به دست آمده با استفاده از نرم افزار SPSS مورد تجزیه و تحلیل قرار گرفت.

Title: اثر اعتیاد والدین به مورفین بر ایجاد و حفظ تقویت طولانی مدت در مسیر نفوذی به ناحیه شکنج دندانه‌ای هیپوکامپ درفرزندان موش صحرایی

http://yafte.lums.ac.ir/article-1-1040-fa.html

مقدمه: شواهد نشان می‌دهد که اعتیاد یکی از والدین به مورفین منجر به اختلال در فرآیند یادگیری و حافظه فرزندان می شود. از آنجایی که تقویت طولانی مدت (LTP) به عنوان یکی از مکانیسم های سلولی یادگیری و حافظه مطرح می باشد، در این مطالعه اثر اعتیاد والدین بر ایجاد و حفظ تقویت طولانی مدت مسیر نفوذی به ناحیه شکنج دندانه‌ای فرزندان موش صحرایی مورد بررسی قرار گرفت. مواد و روش ها: در این مطالعه تجربی 40 سر موش صحرایی ماده و16 سر موش صحرایی نر با مصرف خوراکی مورفین با دوز 32 میلی گرم /کیلو گرم2 بار در روز به مدت 5 روز معتاد شدند و سپس حیوانات جهت انجام جفت گیری به صورتهای ماده معتاد و نر غیر معتاد = گروه تست 1، نر معتاد و ماده غیر معتاد = گروه تست 2 و نر و ماده معتاد = گروه تست 3، گروه شاهد 1 = ماده دریافت کننده دکستروز 5% و نر دست نخورده و گروه شاهد2 = نر دریافت کننده دکستروز 5% و ماده دست نخورده در کنار هم قرار داده شدند. پس از رسیدن فرزندان به سن بلوغ با تحریک الکتریکی مسیر نفوذی، در ناحیه شکنج دندانه ای تقویت طولانی مدت ایجاد شد.میزان تغییرات شیب پتانسیل پس سیناپسی تحریکی (EPSP) و دامنه پتانسیل عمل دسته جمعی نسبت به سطح پایه در فواصل زمانی 120-5 دقیقه پس از تحریک با فرکانس بالا اندازه گیری و نتایج به صورت میانگین ± خطای معیار بیان گردید. نتایج با روش آماری آنالیز واریانس دو طرفه با مدل اندازه گیری مکرر و تست توکی آنالیز شد. نتیجه گیری: با توجه به نتایج این مطالعه می‌توان نتیجه‌گیری کرد که مصرف مورفین توسط والدین می‌تواند منجربه کاهش حفظ تقویت طولانی مدت ایجاد شده در ناحیه شکنج دندانه‌ای در فرزندان گردد که به نوبه خود می‌تواند موجب اختلالاتی در روند‌ حافظه و یادگیری شود. بنابراین باید آگاه کردن افکار عمومی از عواقب اثر اعتیاد بر فرزندان بایستی بیشتر مورد توجه قرارگیرد. یافته ها: نتایج نشان می دهد که شیب پتانسیل پس سیناپسی و دامنه پتانسیل عمل در فرزندان نر و ماده گروه تست 1 و گروه تست 2 در دقایق 60 و 120 پس از تحریک تتانیک به ترتیب بطور معنی داری کمتر از گروه شاهد 1 و2 است. ولی تفاوت معنی داری بین فرزندان نر وماده در هر گروه و بین گروه های تست 1 و تست 2 وجود ندارد.در ضمن در گروه تست 3 بارداری صورت نگرفت.

Title: بررسی تأثیر تریاک بر اسپرماتوژنز مردان معتاد

http://yafte.lums.ac.ir/article-1-1041-fa.html

مقدمه: مواد مخدر باعث تغییر در کارکرد ارگانهای جنسی می شود و معروفترین و قدیمی ترین این مواد تریاک می باشد. اثر این ماده بر اسپرماتوژنز مردان معتاد در منطقه ای بررسی شد که اصلی ترین ماده مورد سو ء مصرف می باشد. مواد و روشها: در این مطالعه مورد- شا هدی مایع سمن 224 نفر که 112 معتاد به تریاک و112 نفر سالم بودند بررسی و یافته ها جمع آوری گردید، سپس توسط آزمونهای آماری مجذور کای و تی تست تجزیه و تحلیل و دو گروه مقایسه شدند. سطح معناداری اختلاف بین دو گروه 05/0p< در نظر گرفته شد. یافته ها: نتایج حاکی از تفاوت آماری معنی دار بین دو گروه از نظر تعداد، شکل، تحرک، ویسکوزیته و زمان سیال شدن سمن بود. بحث و نتیجه گیری: نتایج بررسی نشان میدهد که اعتیاد به تریاک تأثیر پا تولوژیک زیادی بر اسپرماتوژنز دارد و می تواند منجر به ناباروری و کم باروری افراد گردد که احتمالاً به دلیل کاهش سطح تستوسترون در اثر مصرف تریاک و بالطبع کاهش فعالیت بیضه ها و ارگانهای جنسی وابسته می باشد.

Title: بررسی میزان بروز بیداری و بخاطر آوردن حین بیهوشی عمومی

http://yafte.lums.ac.ir/article-1-1042-fa.html

مقدمه: تجربه بیداری و به خاطر آوردن وقایع حین عمل جراحی در طی بیهوشی عمومی همیشه یک مسئله ترس آور هم برای بیماران و هم برای پرسنل بیهوشی بوده است. اگر بیمار حین عمل درد را تجربه کند باعث ایجاد عوارض روحی ـ روانی جدی در وی شده که به شکل اختلال روانی مشخص می گردد و چنانچه بیمار حین عمل جراحی بیدار باشد و قادر به شنیدن بعضی از صحبت های حین عمل باشد احساس ضعف، فلج، درماندگی، اضطراب و مرگ می کند. این مطالعه با هدف تعیین میزان بروز بیداری و بخاطر آوردن حین بیهوشی عمومی انجام گرفته است. بحث و نتیجه گیری: با توجه به اثرات نامطلوب بیداری حین بیهوشی عمومی استفاده از بنزودیازپین ها و مخدرها به عنوان پیش دارو و ضد درد و غلظت های مناسب هوشبری های استنشاقی حین نگهداری بیهوشی عمومی برای پیشگیری این عارضه ضروری به نظر می رسد. یافته ها: اطلاعات بدست آمده نشان داد که 1058 نفر (51/70 درصد) واحدهای پژوهش مرد و 442 نفر (49/29 درصد) زن بودند. در تعداد 900 مورد (60 درصد) از اعمال جراحی از بنزودیازپین ها (دیازپام و میدازولام) به عنوان پیش دارو و در تمامی بیماران از مخدر استفاده شده است. داروی القاء تقریباً در تمامی موارد تیوپنتال سدیم بود. فقط 6 نفر (4/0 درصد) از بیماران به مواردی اشاره کردند که دال بر بیداری و بخاطر آوردن وقایع حین بیهوشی عمومی بود. مواد و روشها: این تحقیق یک مطالعه مقطعی است که در آن 1500 بیمار که جهت اعمال جراحی مختلف به طور انتخابی به اتاق عمل بیمارستان شهدای عشایر خرم آباد مراجعه کرده بودند مورد بررسی قرار گرفتند (تمام بیماران کلاس یک بیهوشی بودند). ابراز گرد آوری اطلاعات پرسشنامه ای بود که حاوی سئوالات لازم جهت بررسی مشکل بود، و 36 ـ 20 ساعت بعد از بیهوشی با مراجعه پرسشگر به بالین بیمار تکمیل می شدند.

Title: مقایسه حساسیت روشهای دید مستقیم میکروسکوپی و کشت در تشخیص تریکوموناس واژینالیس مراجعه کنندگان به مراکز بهداشتی شهر تبریز

http://yafte.lums.ac.ir/article-1-1043-fa.html

مقدمه: تریکوموناس واژینالیس یک پاتوژن شایع در سراسر جهان می باشد که سالانه 180 میلیون نفر را در جهان مبتلا می کند. این بیماری با واژینیت، سرویسیت، وزن کم نوزادان هنگام تولد و زایمان زودرس در زنان حامله مرتبط است. همچنین عاملی برای انتقال عفونت HIV می باشد. تشخیص تریکومونیازیس اغلب با دید مستقیم میکروسکوپی و مشاهده انگل زنده متحرک در ترشحات واژن صورت می گیرد که حساسیت این روش 85-38 % می باشد. روش استاندارد طلائی برای تشخیص انگل، کشت در محیط اختصاصی می باشد که دارای حساسیتی در حدود 95-85% می باشد. در این مطالعه از دو روش کشت و دید مستقیم برای تشخیص تریکوموناس واژینالیس استفاده شده است. مواد و روشها: از 2630 خانم مراجعه کننده به مراکز بهداشتی شهرستان تبریز پس از اخذ رضایت و ثبت علائم بالینی با استفاده از 2 سواپ پنبه دار نمونه برداری شد. سواپ اول بلافاصله در زیر میکروسکوپ از نظر وجود انگل فعال مورد بررسی قرار گرفت و از سواپ دوم جهت کشت نمونه ها در محیط کوپفربرگ استفاده شد. یافته ها: معاینه بالینی از 2630 مراجعه کننده به مراکز بهداشتی نشان داد که 1575 نفر(88/59%) دارای علائم واژینیت و 1055 نفر (11/40%) فاقد علائم بالینی بودند. در بیماران مبتلا به تریکوموناس واژینالیس شایعترین علامت ترشحات واژینال بود. با روش دید مستقیم میکروسکوپی از مجموع 2630 مراجعه کننده، 91 نفر (46/3%) از نظر تریکوموناس واژینالیس مثبت بودند. رشد انگل در محیط کشت در 120 نمونه (56/4%) مشاهده شد. بحث و نتیجه گیری: اگرچه دید مستقیم میکروسکوپی امروزه در دسترس ترین روش جهت تشخیص تریکومونیازیس است اما دارای حساسیت پائینی می باشد. در مطالعه حاضر روش کشت به عنوان استاندارد طلائی در نظر گرفته شد و حساسیت دید مستقیم میکروسکوپی 33/73% و ویژگی آن 88/99% بدست آمد. با توجه به نتایج به نظر می رسد که کشت می تواند روش انتخابی مناسبی برای تشخیص تریکوموناس واژینالیس همراه با روش متداول دید مستقیم باشد.

Title: معرفی یک مورد پلاستیک دفورمیتی فیبولا همراه با شکستگی تیبیا

http://yafte.lums.ac.ir/article-1-1044-fa.html

مقدمه: پلاستیک دفورمیتی عبارتست از تغییر شکل ثابت استخوان بدنبال اعمال نیرو که بر خلاف الاستیک دفورمیتی که با برداشتن نیرو به حالت اولیه برمی گردد، به همان شکل باقی می ماند. این پدیده حالت نادری است و اغلب در اندام فوقانی (ساعد) دیده می شود و غفلت از تشخیص و درمان آن سبب بروز محدودیت حرکت و باقی ماندن دفورمیتی عضو می شود. این حالت بیشتر در بچه ها دیده می شود چون انعطاف استخوانهایشان بیشتر ازبالغین است.برای درمان باید در مورد تشخیص آن دقت شود و حتماً قبل از اصلاح شکستگی پلاستیک دفورمیتی را اصلاح کنیم و تا مدتها پس از اصلاح، با رادیوگرافی کنترل از باقی ماندن اصلاح مطمئن باشیم. نتیجه گیری: همواره به یاد داشته باشیم یکی از علل عدم موفقیت جااندازی بسته شکستگیهای استخوانهای بلند خصوصاً در اندامی که دو استخوان در موازات هم دارد (مثل ساق و ساعد)می تواند پلاستیک دفورمیتی یعنی تغییر شکل ثابت یکی از آنها باشدکه مانع جااندازی دیگری گردیده است. همواره احتمال نیاز به جراحی را به یاد داشته باشیم. معرفی مورد: پسر 8 ساله با شکستگی تیبیا و انحراف فیبولا بدنبال تصادف با موتورسیکلت مراجعه می کند. ابتدا سعی در جااندازی بسته می شود که ناموفق است لذا به کمک جراحی درمان می گردد.

Title: درمان جراحی انسداد کامل آئورت شکمی(Juxtarenal) و گزارش یک مورد

http://yafte.lums.ac.ir/article-1-1045-fa.html

مقدمه: انسداد کامل آئورت شکمی با لنگش متناوب اندام تحتانی دو طرف شروع و در صورت عدم درمان منجر به درد موقع استراحت و زخم ایسکمیک و سرانجام گانگرن عضو می گردد. مهمترین ریسک فاکتورهای بیماری شامل مصرف سیگار، بالا بودن کلسترول و تری گلیسرید، دیابت و افزایش فشار خون می باشند. بیماری با شرح حال و معاینه کامل تشخیص داده می شود و جهت تأیید تشخیص و برنامه درمانی از داپلر رنگی و MRA و یا سی تی آنژیوگرافی استفاده می گردد. نتیجه گیری: درمان انتخابی این بیمار عمل جراحی بای پاس آئورتوفمورال دو طرفه با استفاده از داکرون دو شاخه می باشد. معرفی بیمار: آقای 62 ساله، کشاورز، متأهل با لنگش دو طرفه اندام تحتانی و درد نوک انگشتان هر دو پا و ناتوانی جنسی که از 6 ماه پیش شروع شده بود.
